# Supplementary material for: Inference of neuronal functional circuitry with spike-triggered non-negative matrix factorization
Source: Nat Commun. 2017 Jul 26;8:149. doi: 10.1038/s41467-017-00156-9 (PMC5529558; doi:10.1038/s41467-017-00156-9)
Supplement: Supplementary file 1 — Supplementary Information [file 41467_2017_156_MOESM1_ESM.pdf]

File Name: Supplementary Information
Description: Supplementary Figures and Supplementary References

File Name: Peer Review File
Description:

Supplementary Figure 1

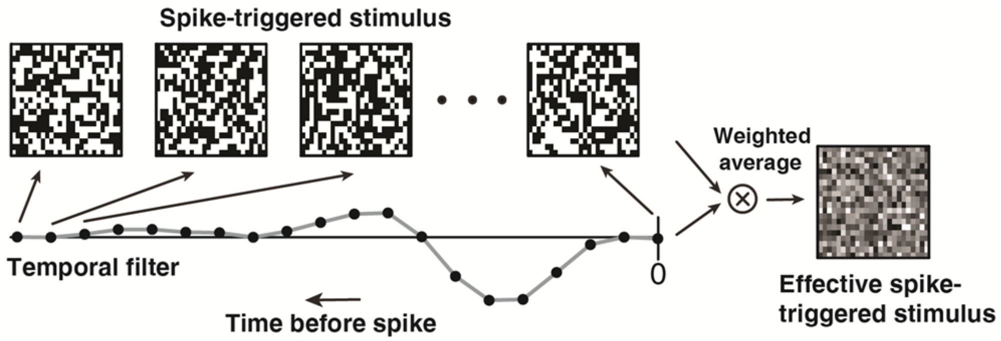

Supplementary Figure 1. Computation of effective spike-triggered stimuli. Each spike-triggered stimulus under spatiotemporal white-noise stimulation is a brief sequence of black-white binary spatial patterns. To integrate out time, the cell's temporal filter, obtained from the spike-triggered average (STA), is used as a set of weights to compute a weighted average of the frames from the spike-triggered stimulus. The weighted average can be thought of as computing for each pixel the scalar product between the temporal filter and the sequence of contrast values. This yields the effective spike-triggered stimulus, a spatial pattern with greyscale pixel values.

Supplementary Figure 2

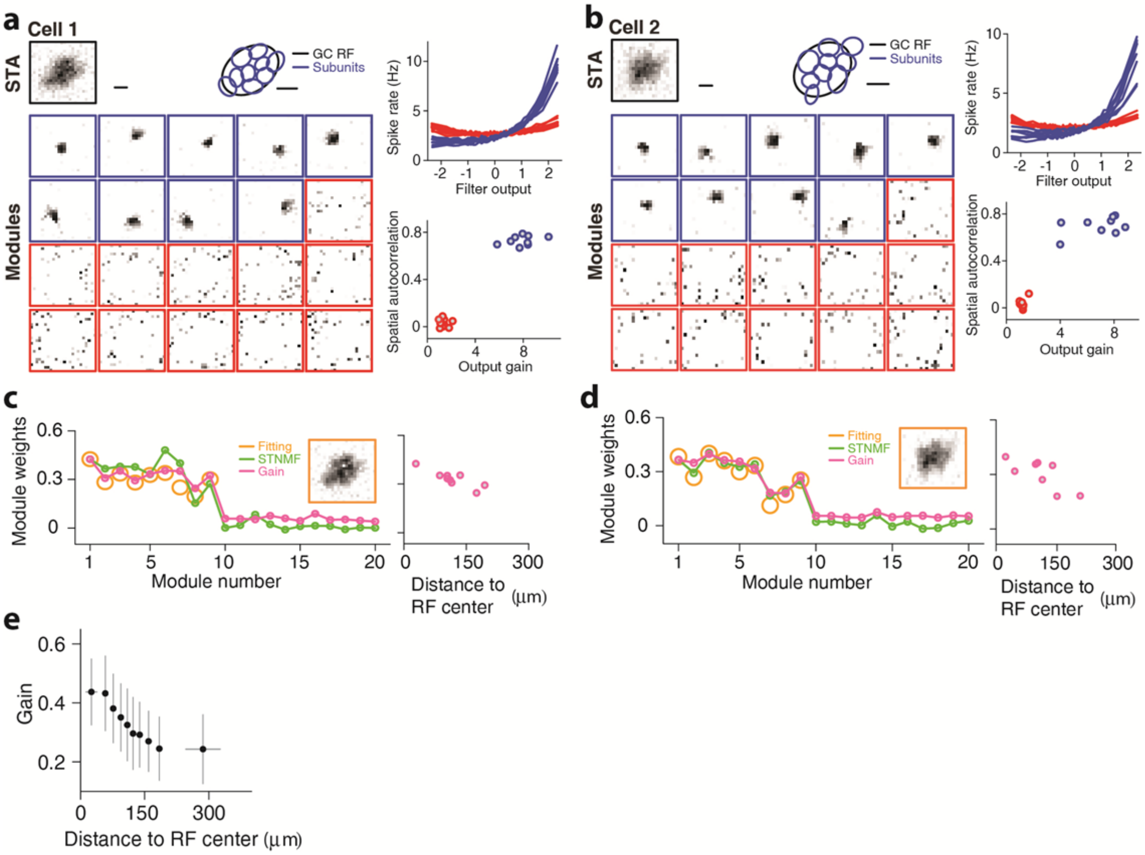

Supplementary Figure 2. Additional examples for the application of STNMF to salamander ganglion cells and analysis of subunit weights. a,b, Subunit analysis of two more sample ganglion cells, analogous to Fig. 1b-d. Scale bars, 100 µm. **c,d**, Module weights according to the computed normalized output gain (magenta), according to the average NMF weights (green), and according to a fit of the spatial receptive field with the detected subunits (orange circles; the inset shows the receptive field as recovered from the fit). Data correspond to the two sample cells shown in (**a**) and (**b**). The STNMF weights have arbitrary scale and were here normalized so that, for the first subunit, the value is the same value as the normalized output gain. The subunits are ordered according to their spatial autocorrelation, from largest to smallest. On the right, the dependence of the normalized output gain on the subunit's distance from the receptive field center is shown. This distance was determined as the distance between the center points of the Gaussian fits. **e**, Average of the dependence of the normalized output gain on the distance from the receptive field center. We determined the subunits and their output gain for 102 ganglion cells from a single retina (*N*=824 subunits) and binned them into ten bins according to the distance from the corresponding receptive field center, so that each bin contained about the same number of subunits. The data show the average normalized output gain and distance for each bin, and error bars correspond to standard deviations.

Supplementary Figure 3

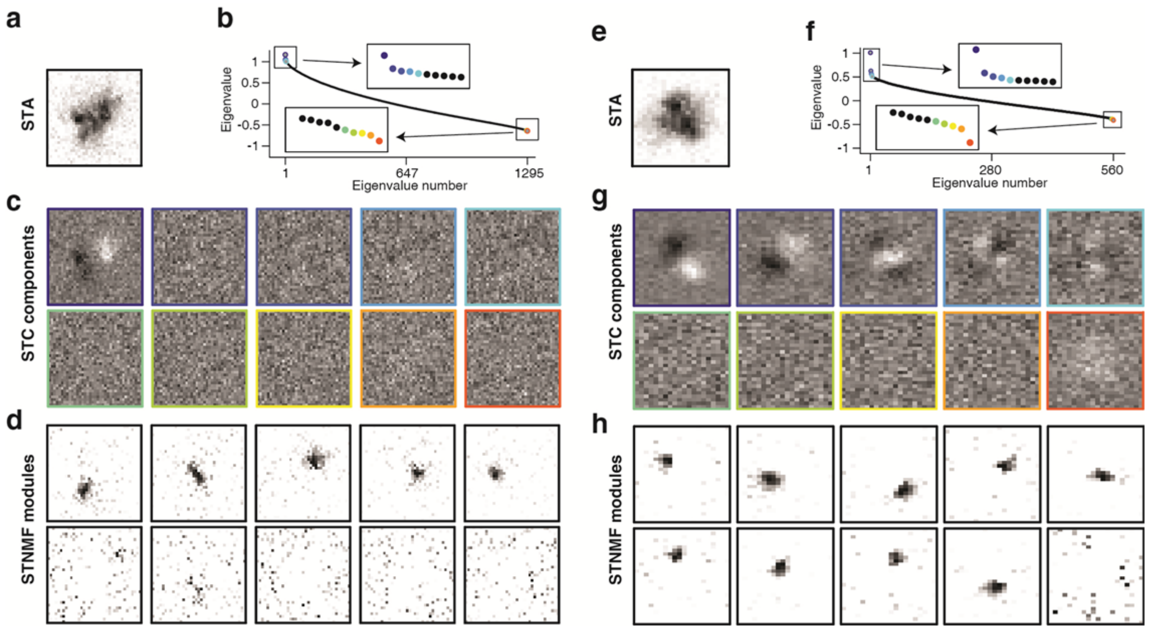

Supplementary Figure 3. Comparison of STNMF and spike-triggered covariance (STC) analysis. We performed STC analysis on the effective spike-triggered stimulus ensembles for individual ganglion cells, using the same spatial region around the receptive field center as for the STNMF analysis. The analysis was performed by computing the covariance matrix of the pixel values across the effective spike-triggered stimulus ensemble and subtracting the prior covariance matrix, which is here given by the identity matrix. We then performed an eigenvalue analysis of this matrix difference. Note that, despite the binary distribution of pixel values in the original stimulus, STC analysis is possible because, owing to the central limit theorem, the temporal weighted averaging yields, to good approximation, normal distributions of pixel values in the effective spike-triggered stimulus ensemble. **a**, Spatial receptive field of a sample cell, obtained as the STA of the effective spike-triggered stimulus ensemble. **b**, Eigenvalue spectrum. Insets show a zoomed-in view of the largest 10 and smallest 10 eigenvalues. Note that there is a broad, continuous distribution of eigenvalues, so that, except for the largest eigenvalue, distinct eigenvalues that signify relevant stimulus features cannot be extracted. This is caused by the high dimensionality of the analyzed stimulus space (here more than 1,000 dimensions), despite the fact that a total of about 9,500 spikes were analyzed. The high dimensionality is necessary to provide sufficient resolution for detecting subunits inside the receptive field.
c, Eigenvectors corresponding to the largest five eigenvalues (top row) and to the smallest five eigenvalues. Here, white and black pixels stand for positive and negative values, respectively.
d, Modules extracted by the STNMF analysis for the same cell. Only the ten modules with the largest spatial autocorrelation are shown. **e-h**, Same as (**a-d**), but for a different sample cell. Here, the STC analysis showed several structured features, though, as opposed to the subunits from the STNMF analysis, not localized in space. About 20,000 spikes analyzed.

Supplementary Figure 4

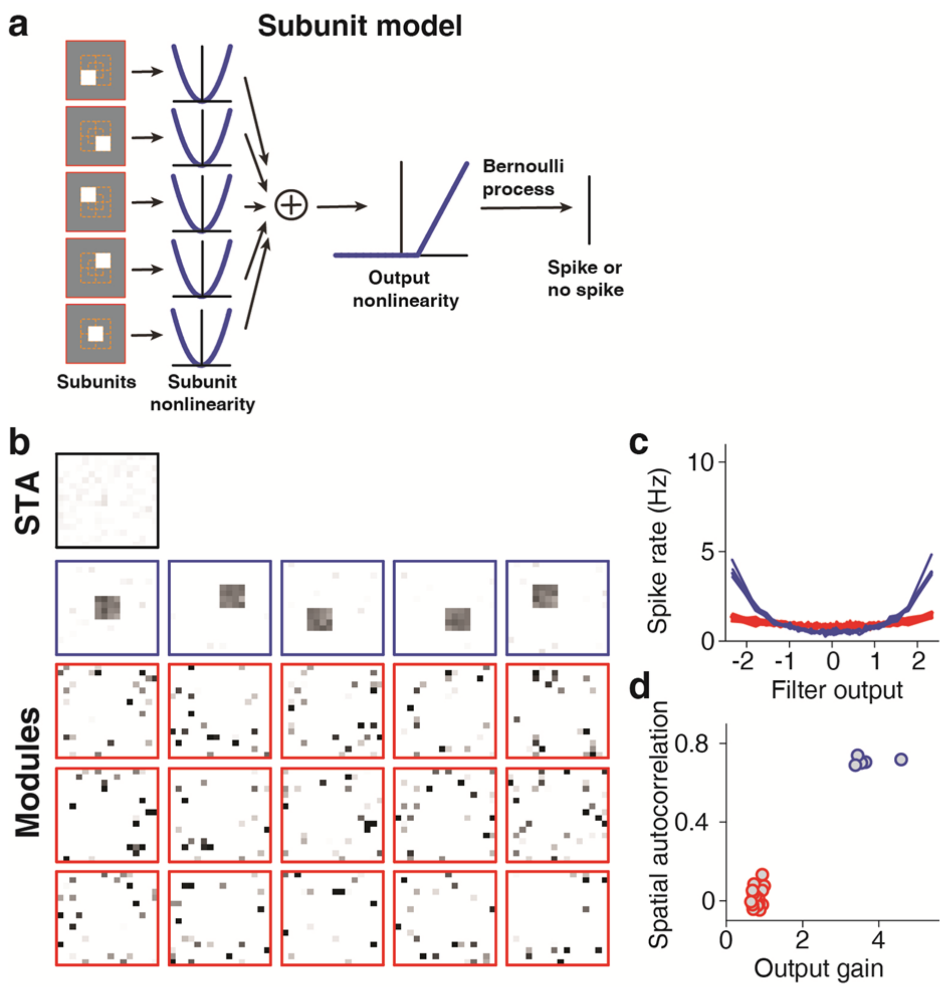

Supplementary Figure 4. Identification of subunits with STNMF for a ganglion cell model with quadratic subunit nonlinearities. a, Model structure. Spatial patterns of 16x16 pixels of Gaussian white noise were used as a stimulus. The model contained five subunits with the same layout as in Fig. 2. For the subunit nonlinearity, however, we here used a symmetric squaring operation. As for the model in Fig. 2, the summed subunit signal then went through a threshold-linear output nonlinearity, and spikes were determined according to a Bernoulli process. **b-d**, STNMF analysis of simulated data as in Fig. 2b-d, using around 3,000 spikes.

Supplementary Figure 5

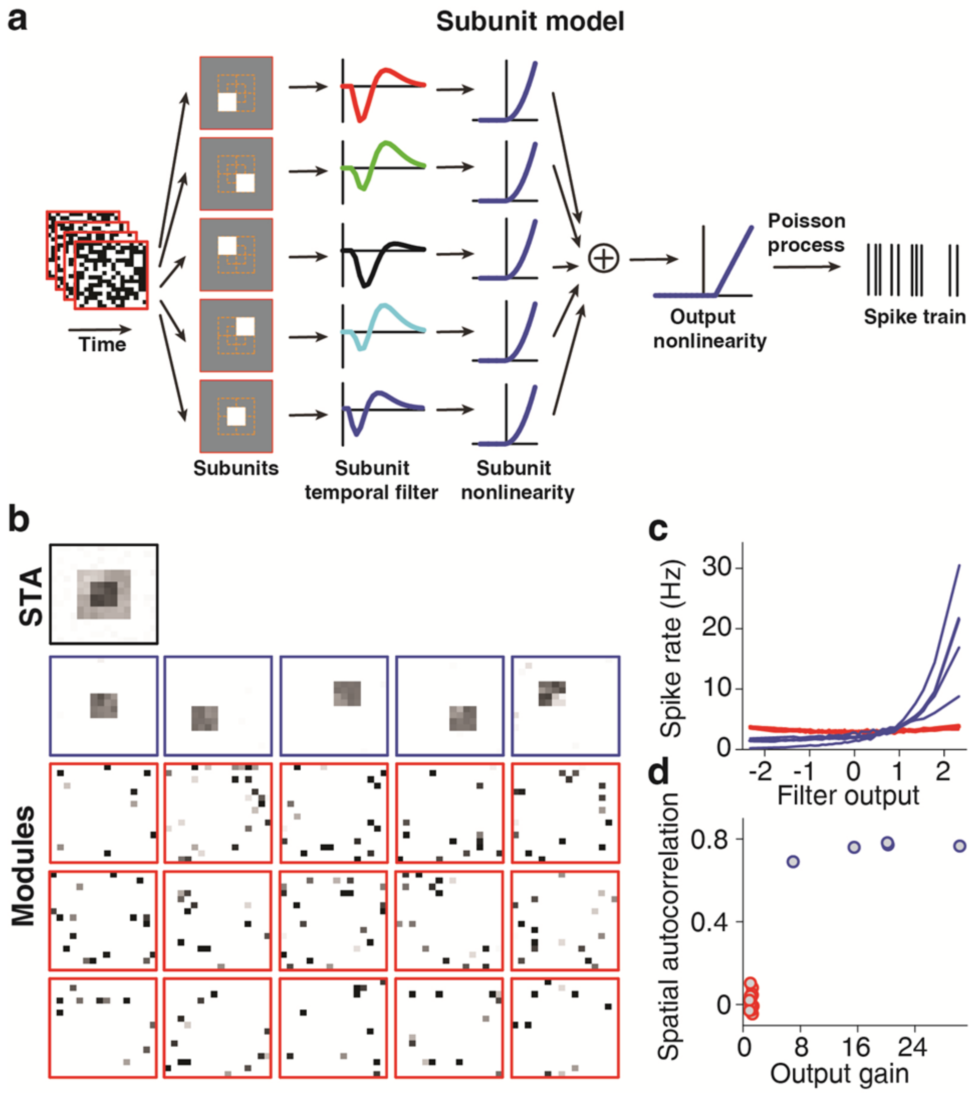

Supplementary Figure 5. Identification of subunits with STNMF for a ganglion cell model with different temporal filters for the subunits. a, Model structure. Binary spatiotemporal white noise with 16x16 pixels was used as a stimulus. The model contained five overlapping subunits as in Fig. 2, each with a different Off-type temporal filter as shown in the plot. As for the model in Fig. 2, each subunit signal is transformed by a threshold-quadratic subunit nonlinearity before summation and application of a threshold-linear output nonlinearity. Spikes were determined according to a Poisson process. **b-d**, STNMF analysis of simulated data as in Fig. 2b-d, using around 6,800 spikes.

Supplementary Figure 6

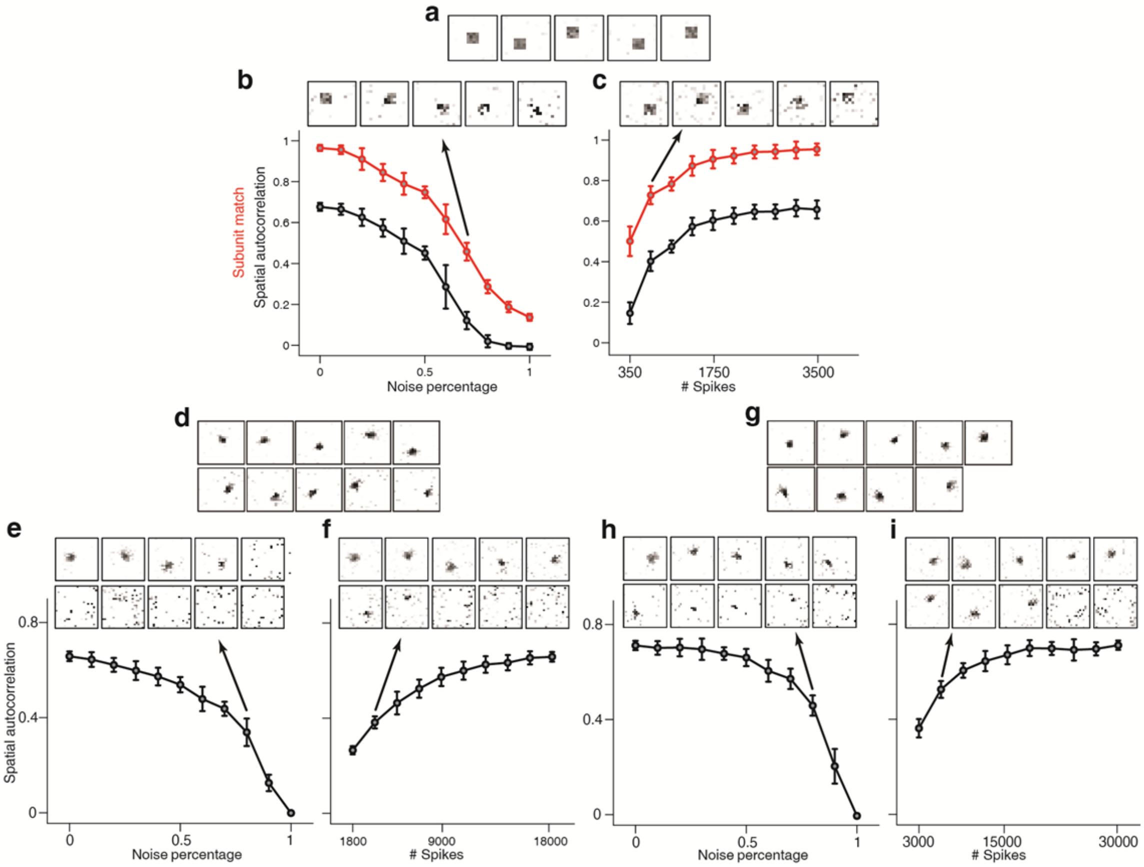

Supplementary Figure 6. Analysis of robustness of subunit reconstruction with respect to noise and number of spikes. a, Reconstructed subunits of the model used in Fig. 2, taking all simulated spikes into account. **b**, Effect of noise, simulated by randomly replacing a fraction of the simulated model spikes (quantified as the “noise percentage”) with spikes at random times. Subunit reconstruction was quantified by the average correlation value between the true subunit and the best matching reconstructed subunit (red) as well as by the average spatial autocorrelation of the five most localized reconstructed subunits (black). Data points and error bars show mean values and standard deviations obtained over 20 repeats of the analysis. The set of subunits on top of the graph shows a sample reconstruction from the noise level indicated by the arrow. **c**, Dependence of subunit reconstruction on available spike number. Limited data sets of specified size were obtained by randomly selecting subsets of the simulated model spikes. Performance of subunit reconstruction was assessed in the same way as in (**b**). **d-f** and **g-i**, Analysis of the effect of noise and spike number for two sample ganglion cells. Performance was assessed, as for the model cell, by the average spatial autocorrelation, taking into account as many reconstructed modules as the number of subunits found for the true data set. Data points and error bars show mean values and standard deviations obtained over 20 repeats of the analysis.

Supplementary Figure 7

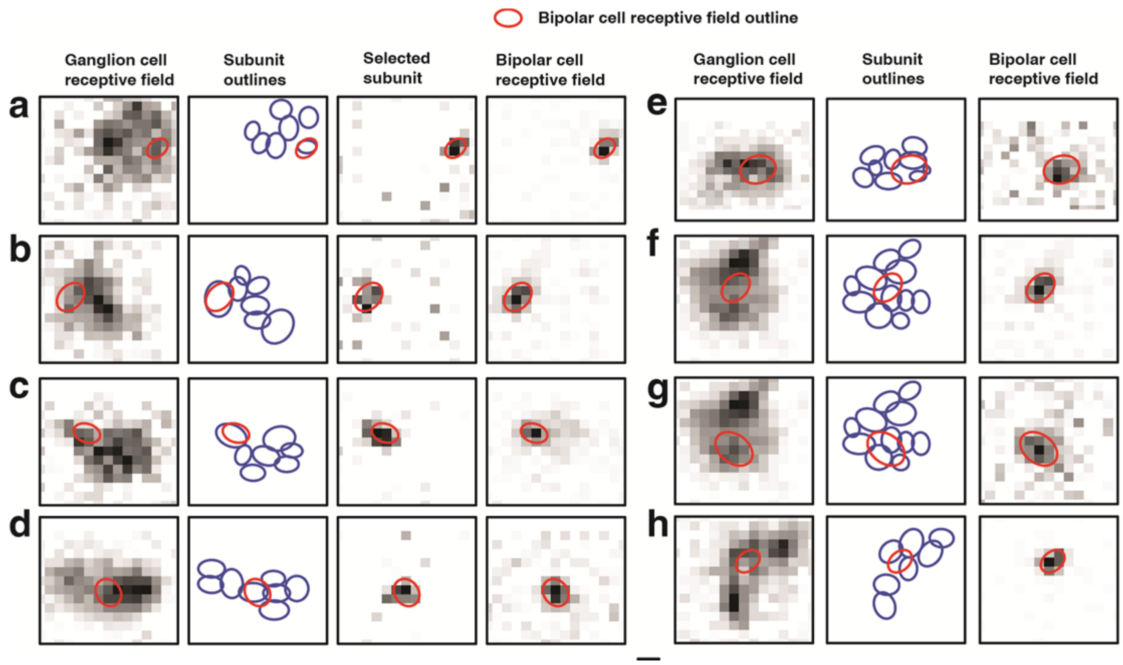

Supplementary Figure 7. Further examples of comparison between recorded bipolar cell receptive fields and extracted ganglion cell subunits. a-d, Four additional examples of bipolar cell-ganglion cell pairs with good matches between the bipolar cell receptive field and one of the reconstructed subunits, shown in the same style as in Fig. 3. **e-h**, Four examples of bipolar cell-ganglion cell pairs for which no subunit matched the bipolar cell receptive field, shown as in (**a-d**), but without a selected subunit. The bipolar cells in (**a**) and (**e**) are the same as in the bottom row and last-to-bottom row, respectively, of Fig. 3, but shown here in comparison to different ganglion cells. (**b**) and (**f**) show the same bipolar cell, once in comparison to a ganglion cell with a matching subunit (**b**) and once in comparison to a ganglion cell with no matching subunit (**f**). (**g**) and (**h**) show bipolar cells for which no match with any subunit of any recorded ganglion cell was found. Scale bar, 100 µm.

Supplementary Figure 8

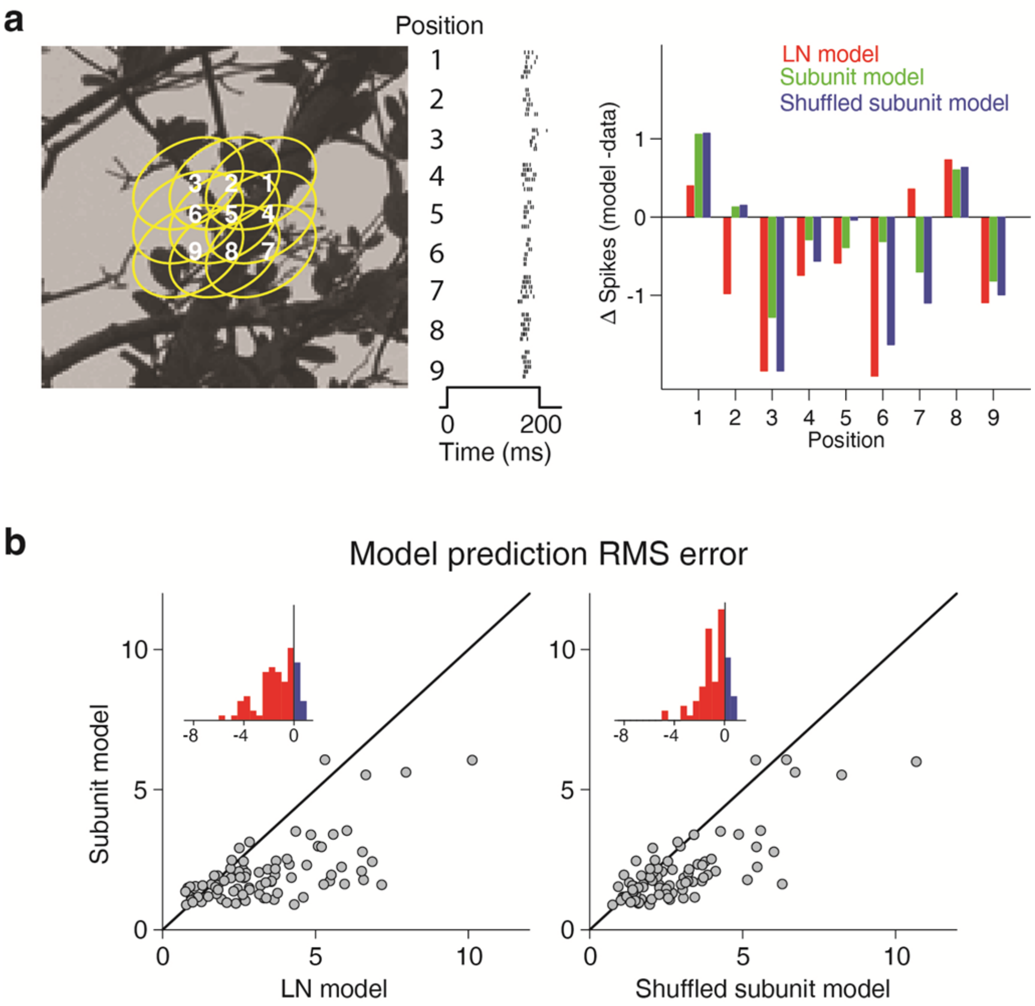

Supplementary Figure 8. Analysis of response predictions with fine-scale shifted natural images. **a**, Responses of a sample ganglion cell. Left: Example of a natural image with receptive field of a ganglion cell at nine positions (ellipses with numbers in the centers), corresponding to the nine slightly shifted presentations of the same image. Center: Raster plots of responses from the sample cell for repeated presentations of the image at the nine positions. Right: Deviations of the three model predictions from the measured mean spike count for each of the nine image positions for the sample cell. The LN model (red), the subunit model (green), and the shuffled subunit model (blue) were similar to the ones used in Fig. 4 in the main text, except that the output nonlinearity was another half-wave rectification (as also used for the subunit nonlinearity), with the slope left as a free parameter, optimized for each model by minimizing the root-mean-square (RMS) error between the data and the prediction. **b**, Comparison of RMS error for the different models. Each data point corresponds to a different combination of analyzed ganglion cell and presented natural image, coming from a total of eleven ganglion cells and ten images from three retinas. Insets show histograms of differences in RMS error. The subunit model generally yielded smaller error values than both the LN model and the shuffled subunit model. Differences between the subunit model and the other two models are significant ($p<10^{-8}$ in both cases; Wilcoxon signed-rank test).

Supplementary Figure 9

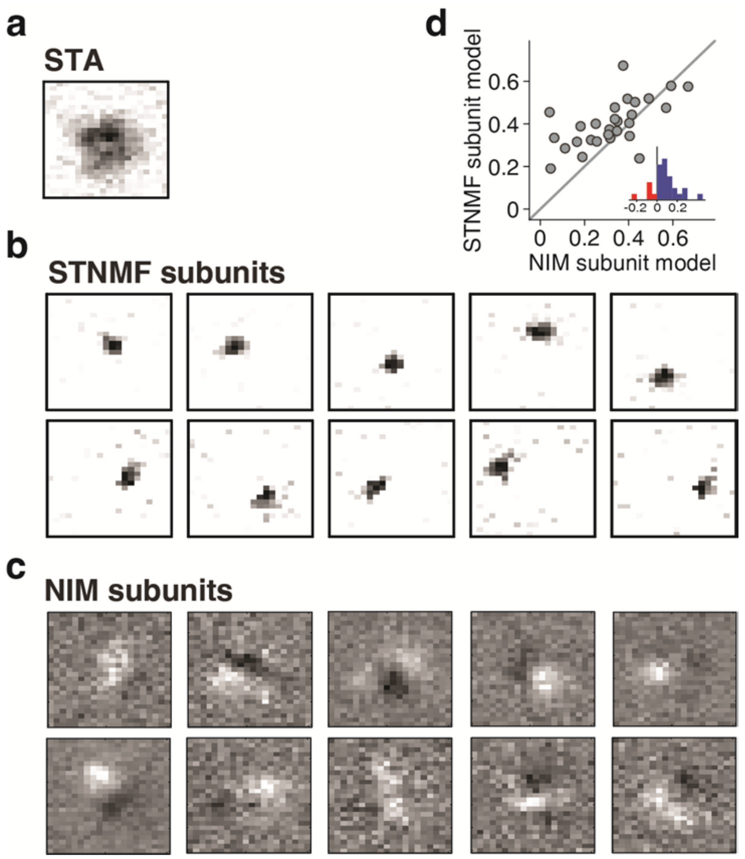

Supplementary Figure 9. Comparison of STNMF with the Nonlinear Input Model (NIM)[1].
a, Spatial receptive field for a sample ganglion cell (same cell as in Fig. 1b-d). **b**, Subunits of the sample cell identified by STNMF. **c**, Subunits obtained from fitting a reduced NIM to the same sample cell. Here, black and white pixels stand for positive and negative values, respectively. **d**, Comparison of response predictions of subunit models with subunits derived from either STNMF or from the reduced NIM fit for the same 28 cells as in Fig. 4c. The analysis was conducted on the recordings under spatiotemporal white-noise stimulation, and performance was measured as the variance explained on the held-out frozen-noise sections. The obtained variance explained was here significantly higher for the STNMF subunit model (p=0.0023; Wilcoxon signed-rank test).

Supplementary Figure 10

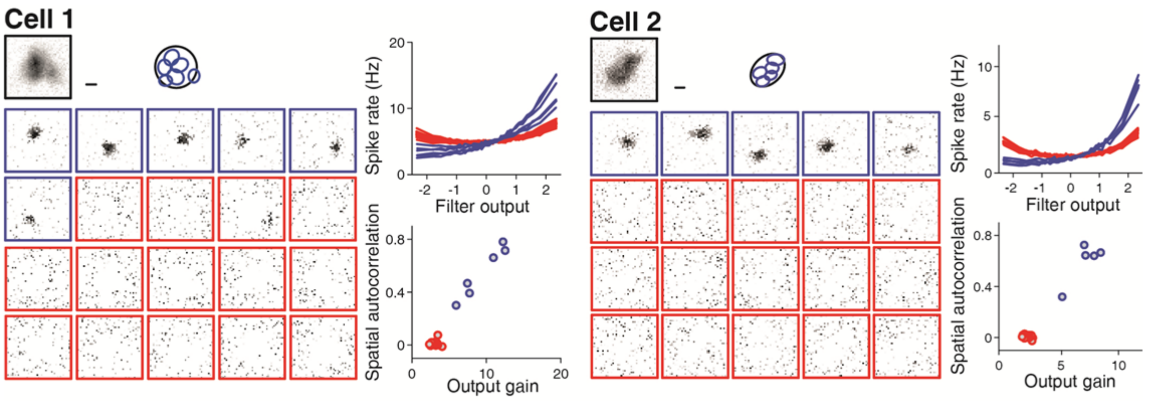

Supplementary Figure 10. Sample application of STNMF to ganglion cell recordings from mouse retina. Reconstructions of subunits from two recorded mouse retinal ganglion cells are shown as in Fig. 1b-d. Scale bars, 100 µm. The applied visual stimulus here was spatiotemporal white noise with a temporal update rate of 15 Hz and a pixel size of 15 µm x 15 µm, half as wide as applied for the recordings from salamander retina. The STNMF method was applied in the same fashion as for the analysis of salamander retinal ganglion cells without any further adjustments or tuning of parameters. Like for the salamander data, STNMF revealed a small number of localized subunits, whose filter output is systematically related to the cell's spiking activity. The extracted subunits could correspond to individual presynaptic bipolar cells. Alternatively, they could represent groups of bipolar cells, in particular since bipolar cells in mouse retina can be coupled by gap junctions, which has been shown to lead to an enlargement of the spatial scale of nonlinear integration[2].

Supplementary References

1. McFarland, J. M., Cui, Y. & Butts, D. A. Inferring nonlinear neuronal computation based on physiologically plausible inputs. *PLoS Comput Biol* **9**, e1003143 (2013).

2. Kuo, S. P., Schwartz, G. W. & Rieke, F. Nonlinear spatiotemporal integration by electrical and chemical synapses in the retina. *Neuron* **90**, 320-332 (2016).
